# Visual-affect-guided spatiotemporal graph modeling of disaster-image diffusion for embodied decision support

**DOI:** 10.3389/fnbot.2026.1889303

**Published:** 2026-09-03

**Authors:** Xiao Zhang, Wei Zhang

**Affiliations:** 1 College of Information Technology and Media, Hexi University, Zhangye, Gansu, China; 2 College of Artificial Intelligence, Shenzhen Technology University, Shenzhen, Guangdong, China

**Keywords:** affective computing, decision support systems, disaster management, graph neural networks, human-robot interaction, online social networks, spatiotemporal phenomena

## Abstract

**Introduction:**

Disaster-response robots and other embodied agents can use social-media imagery as contextual evidence, but existing approaches typically analyze affect at the image level or model diffusion without isolating affect-specific information.

**Methods:**

We encoded disaster images into 128-dimensional continuous visual-affect embeddings representing visual intensity, fear, sadness, and negative valence. These embeddings initialized node states in a time-aware graph neural network whose edges were constrained by observed interaction links, temporal precedence, and spatial reachability. Multi-task heads predicted propagation scale, propagation speed, and spatial-ranking consistency, and a parameter-decomposition module separated affect-associated contributions from structure-associated residuals. Experiments used 4,450 reconstructed image-centered CrisisMMD samples, a common 70/15/15 split, five random seeds, matched baselines, and ablation tests.

**Results:**

The full model achieved an AUC of 0.842 ± 0.011, an RMSE of 0.091 ± 0.007 under the current task definition, and a Kendall tau of 0.730 ± 0.018. Relative to matched ablations, visual affect increased AUC by 0.041, time-aware recurrence reduced RMSE by 33.6%, and spatial constraints increased Kendall tau by 0.089. The RMSE unit must be standardized after the propagation-speed versus propagation-time definition is resolved, as marked in the proof.

**Discussion:**

Visual affect provides an interpretable complementary signal for modeling disaster-image diffusion. The findings do not establish causal effects, cross-platform generalizability, real-time device performance, or closed-loop robot benefit; the framework should therefore be regarded as an upstream social-sensing prior for embodied decision support.

## 1 Introduction

After sudden disasters, social-media images can provide early, geographically distributed evidence of damage, distress, perceived risk, and public attention. For disaster-response robots, mobile sensing platforms, and human-robot teams, these posts should not be interpreted as direct control commands. Instead, they constitute an external social-sensing stream that may complement onboard perception when operators must prioritize locations, allocate sensing effort, or communicate uncertainty. The technical problem addressed here is therefore to transform rapidly changing image streams into a stable spatiotemporal state estimate while maintaining a clear boundary between predictive analytics and physical robot control.

Three limitations motivate the study. First, image-level sentiment classifiers often compress affect into a single discrete label and therefore lose graded variation in intensity, fear, sadness, and negative polarity. Second, diffusion models commonly treat visual content as a generic feature or omit the joint effects of time, geography, and network structure. Third, many system studies report a single performance comparison without a same-protocol baseline hierarchy, module-level evidence, failure analysis, or explicit deployment boundaries. These limitations make it difficult to determine whether visual affect contributes information beyond scene semantics and propagation topology.

Recent studies have advanced visual sentiment analysis for disaster imagery (Hassan et al., 2022; Zhang et al., 2024; Gupta and Roy, 2025; An et al., 2026), dynamic information-diffusion modeling (Chen et al., 2024; Liu et al., 2024; Singh et al., 2024; Muhammad et al., 2026), and multimodal geo-social crisis analysis (Hanny and Resch, 2025; Jalilian and Resch, 2026; Yao et al., 2026). Collectively, these studies establish the value of affective, temporal, spatial, and relational signals. However, they rarely unify affect-specific image embeddings, conservative cascade reconstruction, time-aware graph learning, and interpretable multi-task readout under one controlled evaluation protocol.

This study asks whether affect-specific visual representations provide predictive information beyond generic image semantics and spatiotemporal graph structure for three complementary tasks: propagation-scale classification, propagation-speed regression, and spatial-ranking consistency. The contributions are fourfold: (1) a disaster-aware continuous affect representation that preserves graded visual signals; (2) a conservative image-centered cascade reconstruction procedure with explicit temporal and spatial constraints; (3) a time-aware graph neural network with multi-task prediction and affect-versus-structure parameter decomposition; and (4) a controlled evaluation that combines matched baselines, five-seed reporting, ablation analysis, robustness assessment, error analysis, and computational-scope discussion.

The novelty does not lie in using a graph neural network alone. It lies in testing affect-specific visual information as a traceable node attribute within a reconstructed spatiotemporal diffusion process and in delimiting the conclusions to predictive association. No causal effect of emotion and no closed-loop robot performance are claimed.

## 2 Related work

### 2.1 Visual affect analysis in disaster imagery

Visual sentiment analysis maps image content to affective categories or continuous dimensions. Disaster-focused studies have shown that visual cues can complement textual signals and improve situational interpretation (Hassan et al., 2022; Demir et al., 2024; Gupta and Roy, 2025; Han et al., 2022; Chandrasekaran et al., 2022; Ou et al., 2021; Li et al., 2024b; Ilyas and Sharifi, 2025). More general methods use semantic correlation, user context, cross-attention, or graph-based alignment to improve robustness (Zhang et al., 2024; Liang et al., 2024; An et al., 2026). Their primary endpoint, however, remains image- or post-level sentiment recognition rather than the downstream dynamics of an image-centered diffusion process.

A second issue is representation granularity. Discrete labels are convenient for classification but are poorly suited to modeling gradual changes in urgency or arousal. The present work therefore uses a continuous disaster-aware embedding and evaluates whether it adds predictive value after generic visual semantics and graph structure are controlled.

### 2.2 Dynamic diffusion and spatiotemporal graph modeling

Information-diffusion studies model cascade size, forwarding thresholds, time-varying attractiveness, network influence, and temporal decay (Liu et al., 2024; Zhang et al., 2024; Bakenova et al., 2025; Singh et al., 2024; Atienza-Barthelemy et al., 2025; Zhao et al., 2023; Luo et al., 2023; Sun et al., 2025; Zhang M. et al., 2024; Zhou et al., 2024; Pena et al., 2025). Temporal graph neural networks extend these approaches by updating node states as interactions arrive (Li et al., 2023; Wang et al., 2025; Han et al., 2024; Zhang, 2025). These models provide a strong structural foundation, but content is frequently represented by text or undifferentiated embeddings, so the marginal contribution of visual affect remains unclear.

Crisis datasets also introduce an evidence problem: complete parent–child diffusion trees are rarely available. A model that silently treats inferred edges as observed links can overstate mechanism certainty. The current study addresses this issue by prioritizing explicit interaction evidence, restricting candidate edges by event, time, and spatial consistency, attenuating uncertain links, and retaining isolated records rather than forcing them into unverified cascades.

### 2.3 Multimodal disaster analytics and embodied decision support

Multimodal GeoAI and social-sensing systems combine semantic, temporal, and geographic signals to characterize public response to disasters (Yu and Wang, 2024; Hanny and Resch, 2025; Jalilian and Resch, 2026; Zou and Lv, 2025; Li et al., 2025; Guo and Yang, 2025). Recent work has also modeled retweet behavior with multiple feature modalities (Muhammad et al., 2026) and quantified physical-social disaster impact from multimodal posts (Yao et al., 2026). These systems demonstrate broad analytical value, but their tasks are not designed to isolate affect-specific image information within a dynamic cascade or to produce the three state variables used here.

For embodied decision support, social-media analytics should be treated as an upstream contextual layer. It may help prioritize attention or communication, but it cannot replace onboard sensing, localization, planning, actuation, or operator judgment. This distinction is used throughout the method, results, and conclusion to prevent the analytical model from being presented as a physically validated robot-control system.

### 2.4 Comparative synthesis and research gap

Table 1 organizes the most relevant systems by task, data, modeling focus, and unresolved gap. The comparison is qualitative because the studies use different datasets, labels, sampling strategies, and evaluation protocols. Direct comparison of reported numerical scores would therefore be misleading. The remaining gap is a controlled test of whether affect-specific disaster-image representations improve scale, speed, and spatial-ranking prediction beyond generic visual and structural baselines.

**Table 1:** Guided comparison of recent disaster-image, information-diffusion, and multimodal crisis-analysis studies.

Study	Data and task	Core system and evidence	Gap relative to the present study
Hassan et al. (2022)	Disaster images; visual sentiment analysis	Deep visual models evaluated at the image level	No temporal, spatial, or cascade modeling
Zhai et al. (2024)	Disaster misinformation and correction	Sentiment contagion with social and spatiotemporal proximity	Observational dissemination analysis; no learned image-affect embedding
Chen et al. (2024)	Crisis-information diffusion	Network and temporal diffusion dynamics	No visual-affect representation
Hanny and Resch (2025)	Geo-social disaster posts	Integrated spatiotemporal topic-sentiment GeoAI	Not image-centered cascade prediction
Gupta and Roy (2025)	Disaster-response images	Deep image-analysis framework	No dynamic graph or multi-task diffusion evaluation
Jalilian and Resch (2026)	Four disaster geo-social datasets	Unsupervised multimodal semantic-geographic graph learning	Focuses on clustering/coherence, not cascade evolution
Muhammad et al. (2026)	Twitter retweet prediction	Text, user, engagement, and temporal feature fusion	No disaster-image affect or spatial-ranking task
Yao et al. (2026)	Multiplatform disaster posts	Multimodal impact classification and physi-social indexing	No diffusion graph or affect-versus-structure decomposition
Proposed study	4,450 CrisisMMD image-centered samples	Affect-aware TGNN; scale, speed, ranking; ablation and robustness analysis	Reconstructed cascades; no causal or physical-robot validation

Results reported by different studies were obtained under different experimental settings and are summarized for conceptual comparison, not direct numerical ranking.

## 3 Proposed visual-affect-guided spatiotemporal graph framework

### 3.1 Overall architecture

**Figure 1:**
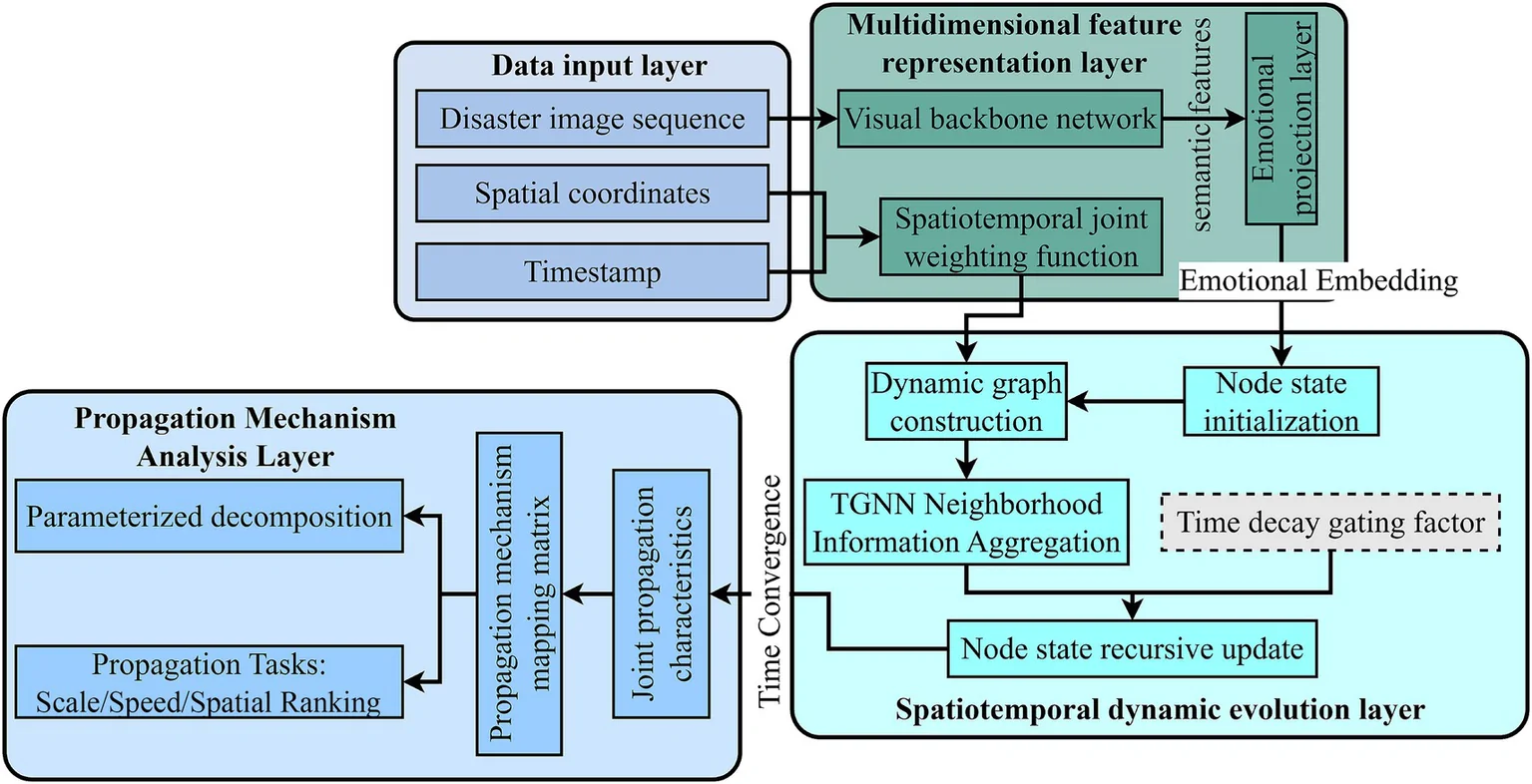
Overall framework for modeling spatiotemporal disaster-information propagation under visual-affect constraints within an embodied decision-support pipeline.

Figure 1 summarizes the revised framework. The model links visual sentiment representation, reconstructed spatiotemporal propagation structure, and mechanism-level analysis. Its intended role is a perception and situation-assessment layer that may inform downstream neurorobotic or physical-AI systems; it is not presented as a complete robot planning architecture.

The framework uses disaster images, timestamps, user-interaction records, and spatial coordinates as unified inputs. Image sentiment embeddings are produced by a visual backbone followed by an emotion-projection layer. A dynamic graph is then formed under temporal-order and spatial-reachability constraints, and a time-aware graph neural network updates node states as the cascade evolves. The output representation is mapped to propagation-scale, speed, and spatial-ranking indicators. In an embodied interpretation, these indicators are contextual cues for risk-aware prioritization and human-robot communication, not substitutes for onboard perception, planning, or actuation.

### 3.2 Disaster-aware visual affect representation

Raw image pixels are unsuitable as stable variables for diffusion analysis because they mix scene category, damage content, color, composition, and affect. Each image is therefore mapped first to a generic visual representation and then to a lower-dimensional continuous affect embedding. This two-stage design permits a direct comparison between generic visual semantics and affect-specific information.

Images are resized to 224 × 224 pixels and normalized using the statistics of the pretrained backbone. The backbone output is passed through a two-layer projection head with dropout and mapped to a 128-dimensional affect embedding. Supervision is based on normalized visual intensity, fear, sadness, and negative-polarity scores. All calibration thresholds are estimated on the training or validation data and are not fitted to the test subset.

Let *I_i_* denote the *i*-th disaster image and let the visual-backbone parameters be *θ_v_*. The high-level visual representation is defined by Equation 1.

$$h_{i}=f_{\theta_{v}}(I_{i})\;$$ (1)

In Equation 1, *h_i_* is a *d_h_*-dimensional representation that preserves scene semantics. Because *h_i_* still mixes disaster category and affective evidence, a dedicated projection head is required before the feature enters the diffusion graph.

The affect projection applies a learned linear transformation followed by a nonlinear activation, as defined in Equation 2.

$$e_{i}=\sigma (W_{e}h_{i}+b_{e})$$ (2)

In Equation 2, *W_e_* and *b_e_* are the projection parameters, and *e_i_* is the continuous affect embedding. Unlike a single categorical label, *e_i_* preserves graded variation that can be compared across disaster types and propagated through the graph.

The projection space is regularized by an affect-regression objective using the normalized four-dimensional target vector *y_i_*. The loss is defined in Equation 3.

$$L_{\mathrm{emo}}=\frac{1}{N}\sum_{i=1}^{N}{\left\|W_{r}e_{i}-y_{i}\right\|}_{2}^{2}$$(

Equation 3 penalizes disagreement between the projected embedding and the affect targets over N training samples. This constraint reduces irrelevant scene variation and makes distances in the embedding space more interpretable for subsequent propagation modeling.

After optimization, the affect embeddings form a shared coordinate system across disaster categories. Their role is deliberately limited: they encode model-estimated visual affect, not the internal emotional state of an individual viewer and not a causal mechanism of sharing behavior.

**Figure 2:**
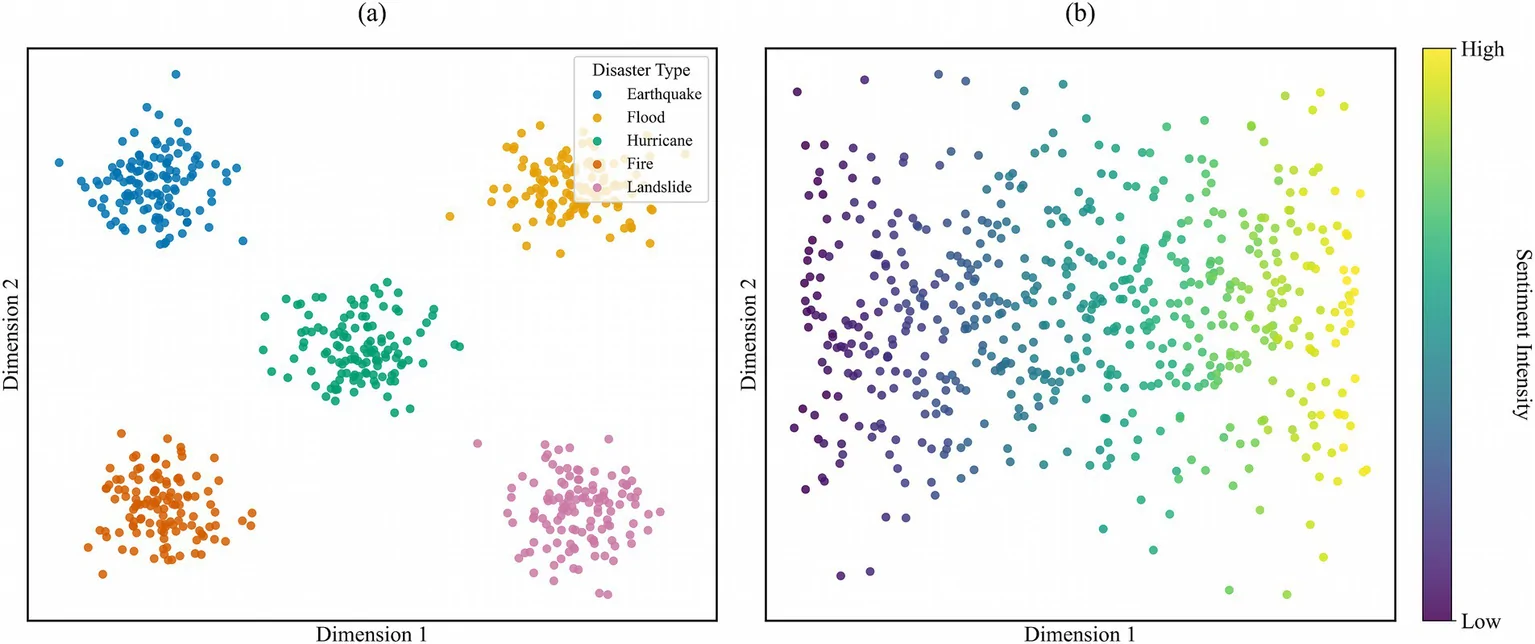
Distribution of samples before and after affect-specific projection. **(a)** Generic visual semantic space; **(b)** continuous visual-affect embedding space.

Figure 2 illustrates the intended distributional effect of the projection by contrasting scene-dominated visual semantics with the more continuous organization of affect intensity.

The generic visual space clusters strongly by disaster category, whereas the projected space orders samples more continuously by affect intensity. This transformation is expected to help the temporal graph distinguish cascades with similar scene content but different affective profiles. The visualization is illustrative and does not by itself establish downstream predictive benefit; that contribution is tested through the matched baseline and ablation experiments in Section 5.

### 3.3 Spatiotemporal cascade reconstruction

Disaster-image diffusion depends on path order, elapsed time, and geographic movement. An affect embedding used without a graph would ignore cascade dependency, whereas a graph without affective attributes would omit part of the content signal. The model therefore places the affect embedding in a directed dynamic graph whose edges are weighted by temporal and spatial plausibility.

Let *V* be the set of image-centered propagation records. Each node *v_i_* has an affect embedding *e_i_*, timestamp *t_i_*, and spatial coordinate *s_i_*. A directed edge from *v_i_* to *v_j_* is eligible only when interaction evidence or conservative reconstruction rules support temporal precedence and spatial reachability. Its joint weight is defined in Equation 4.

(4) $$w_{\mathrm{ij}}=\exp(-\alpha |t_{j}-t_{i}|)\exp(-\beta {\left\|s_{j}-s_{i}\right\|}_{2})$$ 3)

In Equation 4, alpha and beta control temporal and spatial attenuation. Long time gaps and large geographic jumps receive smaller weights, reducing the influence of weak or implausible reconstructed links (Chen et al., 2024; Cheong and Babcock, 2021).

CrisisMMD does not contain complete ground-truth diffusion trees. Explicit retweet, quote, reply, or reference relations are therefore used whenever available. When a parent–child link is missing, a candidate edge is admitted only when the records share an event context, satisfy temporal precedence, and fall within the spatial threshold. Links supported by partial evidence are down-weighted, and isolated records remain single-node cascades rather than being forced into unverified chains.

The reconstructed graph at time *t* is denoted *G_t_*. The initial node state is defined in Equation 5, which makes the affect embedding directly traceable at the start of temporal message passing.

$$x_{i}^{0}=e_{i}$$ (5)

Equation 5 initializes *x_i_^0^* with the affect representation. Structural and temporal information enters through subsequent neighborhood aggregation rather than being conflated with the initial affect signal.

For implementation, the evolving graph is represented as a temporal adjacency tensor. The element at row i, column j, and time t is defined in Equation 6.

$$A_{\mathrm{ij}}^{t}=\{\begin{array}{ll} w_{\mathrm{ij}}, & e_{\mathrm{ij}}\in {ℰ}_{t},\\ 0, & \text{otherwise}, \end{array}$$ (6)

Equation 6 sets the adjacency value to the weighted edge when the relation is active and to zero otherwise. The representation preserves temporal order and supports sparse message passing over each observation window.

The reconstruction should be interpreted as an analytical approximation to available platform evidence. All conclusions that depend on graph topology are therefore conditional on this reconstruction procedure, and the limitation is carried into the discussion and conclusion.

### 3.4 Time-aware graph neural network

The dynamic adjacency tensor describes when and where information may flow but does not produce task-level quantities. A time-aware graph neural network (TGNN) recursively updates node states so that propagation depth, temporal rhythm, and spatial reach are represented in a common trajectory rather than reduced to static cascade statistics.

At discrete time *t*, the hidden state of node *v_i_* is *x_i_^t^*. Neighbor states from the preceding step are aggregated with the active edge weights, as defined in Equation 7.

$$m_{i}^{t}=\sum_{j}A_{\mathrm{ji}}^{t}Ux_{j}^{t-1}\;$$ (7)

In Equation 7, U maps the previous hidden state before weighted aggregation. Information can enter node i only through edges that satisfy the specified event, temporal, and spatial constraints.

A time-decay gate controls the influence of historical activity and prevents long-inactive nodes from receiving unrealistically large updates. The gate is defined in Equation 8 (Li and Li, 2022; Li et al., 2024a).

$$\gamma_{i}^{t}=\exp(-\lambda \Delta t_{i}^{t})$$ (8)

In Equation 8, the elapsed interval since the most recent activation determines the gate magnitude through decay parameter lambda. The gate therefore makes the recurrence sensitive to diffusion tempo rather than treating unequal intervals as equivalent.

The gated neighborhood message and the node self-state are combined through the recursive update in Equation 9.

$$x_{i}^{t}=\tanh(\gamma_{i}^{t}m_{i}^{t}+Wx_{i}^{t-1})$$ (9)

Equation 9 retains the previous state through W while incorporating the gated neighborhood message. Across time, the resulting hidden-state sequence encodes both structural progression and temporal attenuation.

Node states are pooled over the observation window to obtain the cascade-level representation z in Equation 10.

$$z=\frac{1}{T}\sum_{t=1}^{T}\sum_{i}x_{i}^{t}$$ (10)

The pooled representation is shared by the classification, regression, spatial-ranking, and mechanism-decomposition heads. This common representation enables task comparisons without changing the underlying graph encoder.

**Figure 3:**
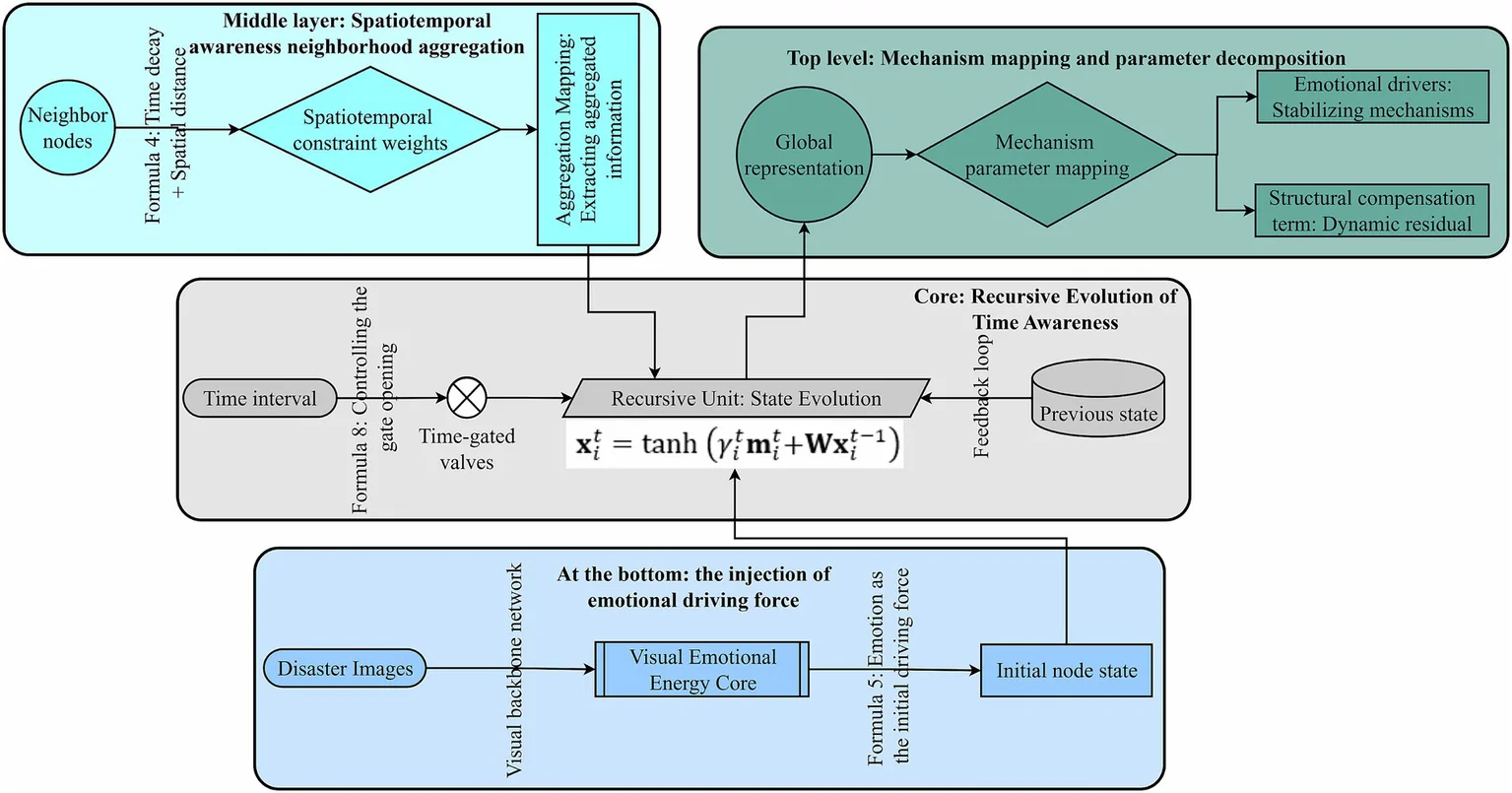
Schematic diagram of the hierarchical computational flow and mechanism decomposition of the time-aware graph neural network (TGNN).

Figure 3 illustrates the hierarchical computational flow of the time-aware graph neural network. The bottom layer embeds visual sentiment into the initial node state, establishing an affective context for diffusion behavior. The middle layer uses a spatiotemporal joint-weight matrix to aggregate neighborhood information and characterize attenuation across physical distance and time. The core unit adjusts recursive evolution through a time-gating factor, and the top layer connects the global representation to the mechanism-parameter space for quantitative decomposition.

### 3.5 Multi-task readout and mechanism decomposition

The pooled TGNN representation is latent. To make it operationally interpretable, separate heads map it to propagation-scale probability, propagation-speed estimate, and spatial ordering. A parameter-decomposition constraint then summarizes the portion associated with the affect embedding and the residual portion associated with graph structure.

Let z denote the pooled cascade representation and y the vector of task outputs. The linear readout is defined in Equation 11.

$$\hat{y}=W_{p}z+b_{p}$$ (11)

In Equation 11, *W_p_* and *b_p_* map the common representation to the task space. The output is predictive; it does not by itself identify a causal pathway.

To prevent the interpretation layer from attributing all variation to topology, the mechanism objective regularizes the task mapping against an affect-associated mapping. The objective is given in Equation 12.

$$L_{\text{mech}}={\left\|\hat{y}-y\right\|}_{2}^{2}+\eta \;{\left\|W_{p}-W_{e}\right\|}_{F}^{2}$$ (12)

Equation 12 combines task error with a Frobenius-norm penalty controlled by eta. The regularizer preserves an explicit affect-associated component while allowing the structural residual to capture cascade-specific effects (Zhang et al., 2022; Xu et al., 2025).

After optimization, the mechanism matrix is decomposed into affect-associated and structure-associated terms, as shown in Equation 13.

$$W_{p}=W_{s}+W_{d}$$ (13)

The decomposition in Equation 13 is a model summary under the observed data distribution. It should not be interpreted as proof that visual affect causes diffusion or that the two terms are uniquely identifiable outside the adopted regularization scheme.

A stability loss is added to discourage large changes in the affect-associated mechanism parameters across observation windows. The loss is defined in Equation 14.

$$L_{\text{stab}}=\sum_{k}{\left\|{W_{s}}^{\left(k\right)}-{\bar{W}}_{s}\right\|}_{F}^{2}$$ (14)

Equation 14 penalizes deviation from the cross-window mean. This regularization supports more stable comparison across event phases, while the subsequent SHAP analysis provides a complementary local explanation of how affect dimensions contribute to model predictions over time.

### 3.6 Training and inference procedure

Training is organized to keep representation learning, graph reconstruction, model selection, and final evaluation separate. Algorithm 1 summarizes the procedure and identifies the points at which leakage controls are applied.

ALGORITHM 1 Training and inference procedure.
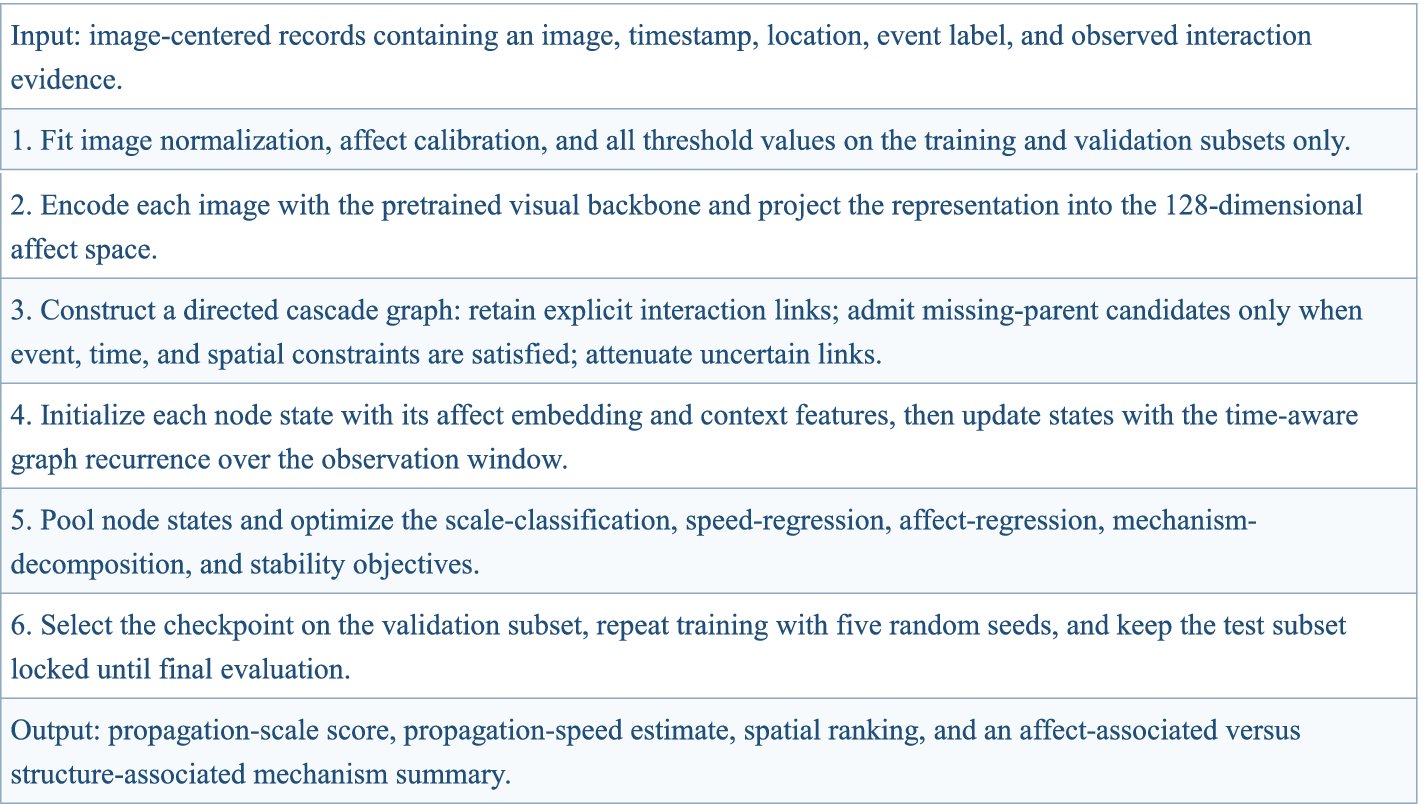

During inference, a new record can be encoded and inserted into the active graph window without recomputing all historical node representations. This supports incremental use, although empirical end-to-end latency was not measured in the present study and no real-time deployment claim is made.

## 4 Experimental design and comparative protocol

### 4.1 Dataset, screening, and preprocessing

Experiments use the CrisisMMD multimodal disaster dataset (Alam et al., 2018), which contains disaster-related images and associated social-media metadata. Because the source data do not provide a complete diffusion tree for every image, image-centered propagation samples were reconstructed from timestamps, event labels, interaction identifiers, and available geographic information. After screening, the analytical dataset contained 4,450 image-centered samples from earthquake, flood, hurricane, fire, and landslide events.

A record was retained only when the image, timestamp, event label, and minimum propagation-order evidence were available. Duplicate images, invalid timestamps, missing key fields, and duplicate images, invalid timestamps, and records with missing key fields were removed. Images were resized to 224 × 224 pixels. Temporal inputs included absolute time and relative intervals. Geographic coordinates were projected into a common two-dimensional system and normalized within event.

Each image defines the root observation of an image-centered cascade. Explicit forwarding or referencing metadata generated directed edges when available. Missing-parent candidates were considered only under shared event context, temporal precedence, and spatial reachability; uncertain candidates received attenuated weights. This procedure increases analytical coverage but does not convert inferred links into ground truth.

Table 2 summarizes the screened dataset by disaster type. The same screened records were used for all controlled baseline and ablation comparisons.

**Table 2:** Statistical characteristics of the reconstructed disaster-image dataset.

Disaster type	Image samples	Average propagation depth	Average spatial-coverage radius (coordinate units)
Earthquake	1,245	4.32	186.7
Flood	1,018	3.87	164.5
Hurricane	892	4.05	201.3
Fire	764	3.41	149.8
Landslide	531	3.26	132.4

The spatial-radius values use the projected coordinate units adopted during preprocessing. More importantly, Table 2 documents the event imbalance and the reconstructed nature of the samples. The dataset supports controlled internal comparison, but external validity must be tested on independent events and platform-native cascade logs.

### 4.2 Tasks and evaluation metrics

Three tasks are evaluated in the same representation space. Propagation-scale prediction is a binary classification problem within a fixed observation window. The true class is *y_i_* and the model score is *s_i_*. Discrimination is measured by the area under the receiver operating characteristic curve, defined in Equation 15.

$$\mathrm{AUC}=\int_{0}^{1}\mathrm{TPR}(u)\text{dFPR}(u)$$ (15)

The AUC integrates true-positive rate against false-positive rate over all thresholds and is therefore less dependent on a single decision threshold than accuracy. Higher values indicate better separation between high- and low-propagation cascades.

Propagation speed is defined as the average change in cascade depth per hour. Let *v_i_* be the observed speed and *v̂_i_* the estimate. Regression error is measured by RMSE in Equation 16; MAE and R-squared are additionally reported for the intensity-group analysis.

$$\text{RMSE}=\sqrt{\frac{1}{N}\sum_{i=1}^{N}{\left({\hat{v}}_{i}-v_{i}\right)}^{2}}$$ (16)

RMSE penalizes large errors and is expressed in h^-1^ under the adopted task definition. Lower RMSE and MAE indicate closer agreement, whereas higher R-squared indicates that the model explains more of the observed speed variation.

Spatial-diffusion consistency measures whether the model preserves the observed geographic ordering of cascade nodes. Let *π_i_* be the observed order and *π̂_i_* the predicted order. Kendall rank correlation is defined in Equation 17.

$$\tau =\frac{C-D}{\frac{1}{2}M(M-1)}$$ (17)

Concordant and discordant node pairs determine Kendall tau. The metric emphasizes relative geographic ordering rather than absolute coordinate error, which is appropriate for prioritization and ranking applications.

Together, the three metrics test complementary properties: discrimination of high-reach cascades, numerical estimation of diffusion tempo, and preservation of spatial order. No single metric is treated as sufficient evidence for system usefulness.

### 4.3 Baseline models and ablation design

Four controlled systems were trained under the same reconstruction, split, and evaluation protocol. IC-FR combines an independent-cascade formulation with manually derived cascade features. ST-GCN learns from discrete spatiotemporal graph slices without visual input. Visual-TGNN adds generic image embeddings to the time-aware graph. Sentiment-TGNN, the proposed full system, replaces the undifferentiated visual signal with the disaster-aware affect embedding while retaining the same graph encoder and task heads.

The baseline hierarchy isolates the incremental value of structural dynamics, generic visual semantics, and affect-specific representation. The ablation study further removes visual affect, temporal recurrence, spatial constraints, or both spatiotemporal constraints while keeping the remaining architecture unchanged. This matched design is more informative than cross-paper numerical comparison because all systems receive the same data split and preprocessing.

### 4.4 Implementation details, reproducibility, and statistical analysis

The dataset was stratified into training, validation, and test subsets at approximately 70/15/15 while preserving disaster type and affect-intensity bins. The TGNN hidden state and affect embedding were both 128-dimensional. Adam optimization used a learning rate of 1e-3, weight decay of 1e-5, batch size 32, and early stopping after 15 validation epochs without improvement. Table 3 consolidates the reproducibility settings.

**Table 3:** Implementation and reproducibility settings.

Component	Setting	Value or rule
Input	Image size	224 × 224 pixels
Representation	Affect embedding	128 dimensions
TGNN	Hidden-state dimension	128
Optimization	Optimizer/learning rate	Adam/1e-3
Regularization	Weight decay/dropout	1e-5/projection-head dropout
Training	Batch size/early stopping	32/15 validation epochs
Partition	Train/validation/test	Approximately 70%/15%/15%, stratified
Robustness	Independent runs	Five random seeds
Model selection	Selection criterion	Validation AUC and validation RMSE; test set locked

All main quantitative results are reported as mean ± standard deviation across five random seeds when repeated-run values are available. The low-versus-high intensity regression comparison additionally reports a prespecified *p*-value. Statistical significance is not asserted for comparisons lacking paired raw predictions; instead, effect magnitudes and seed-to-seed variability are reported explicitly. This distinction prevents descriptive differences from being overstated.

The implementation is organized into data screening and graph reconstruction, visual-affect encoding, temporal graph learning, and evaluation. The test subset remained locked during preprocessing calibration, threshold selection, hyperparameter choice, and early stopping. Processed reconstruction metadata and code can be released on reasonable request, subject to the use conditions of CrisisMMD.

### 4.5 Experimental workflow and computational complexity

**Figure 4:**
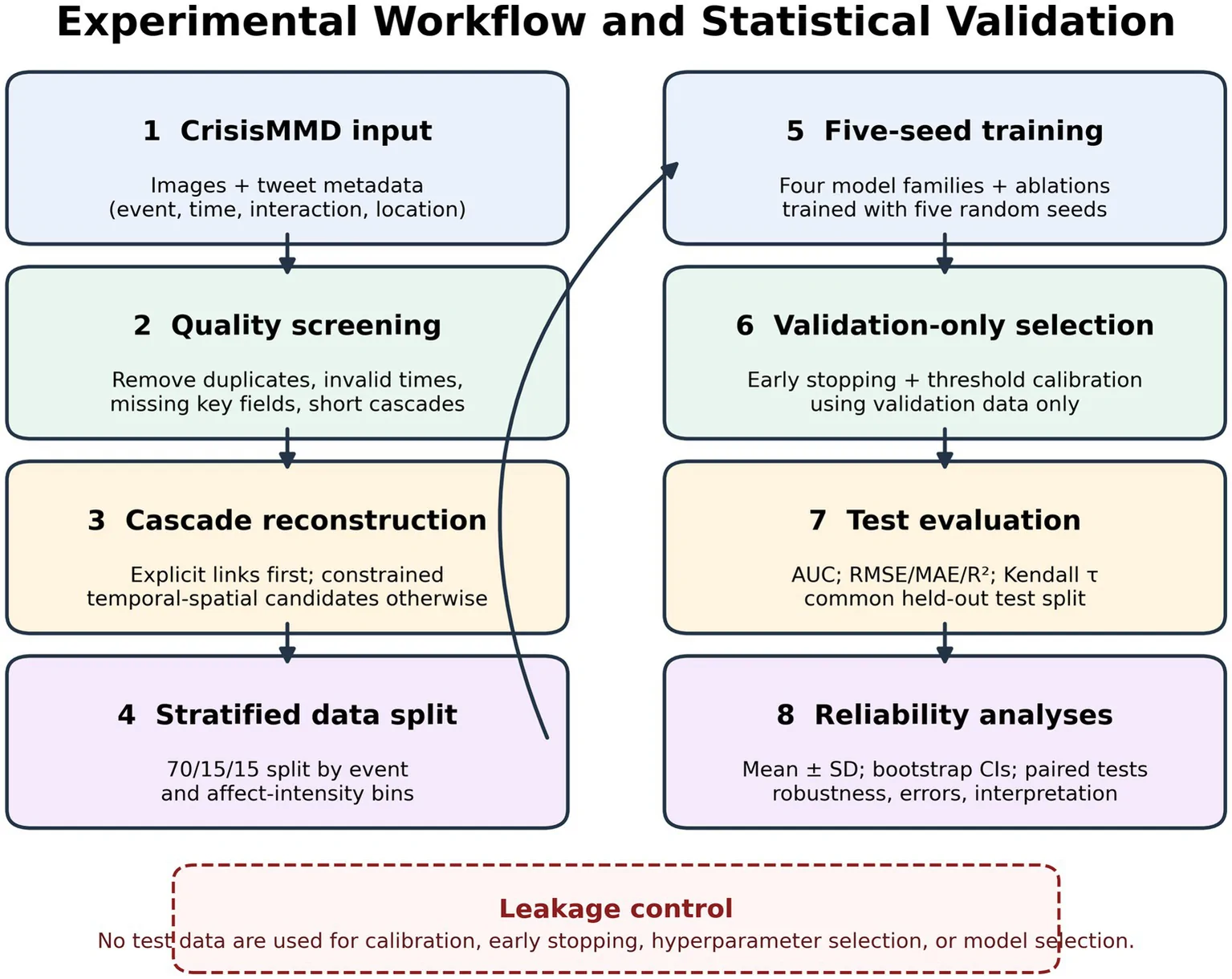
Experimental workflow, data-partition boundaries, and leakage controls for the controlled comparison.

Figure 4 makes the evaluation sequence and leakage controls explicit. Training-only transformations precede graph learning; validation data are used for checkpoint and threshold selection; and the test subset is accessed only after the model configuration is fixed.

Theoretical complexity is summarized in Table 4. Let *d* be the hidden dimension, *V_t_* the active nodes, *E_t_* the active edges in a time window, and *C_v_* the fixed visual-encoder cost per image. Sparse graph updates scale with active edges rather than the square of all records, which permits incremental processing.

**Table 4:** Theoretical computational cost and deployment interpretation.

Component	Time complexity	Memory	Interpretation
Visual encoding	O(N *C_v_*)	O(N d)	Computed once per image; dominates when the backbone is not cached
Graph construction	O(|*E_c_*|) after candidate generation	O(|E|)	Sparse, evidence-constrained reconstruction
TGNN update	O(|*E_t_*| *d* + |*V_t_*| *d*^2^)	O(|*V_t_*| *d* + |*E_t_*|)	Supports windowed or incremental message passing
Readout and heads	O(|*V_t_*| *d* + *d K*)	O(d K)	Small additional cost for K task outputs

Table 4 describes algorithmic feasibility, not measured real-time performance. Training time, device-level latency, memory consumption, and robot-integration overhead were not benchmarked and remain necessary components of future deployment validation.

## 5 Results and discussion

### 5.1 Overall performance comparison

**Figure 5:**
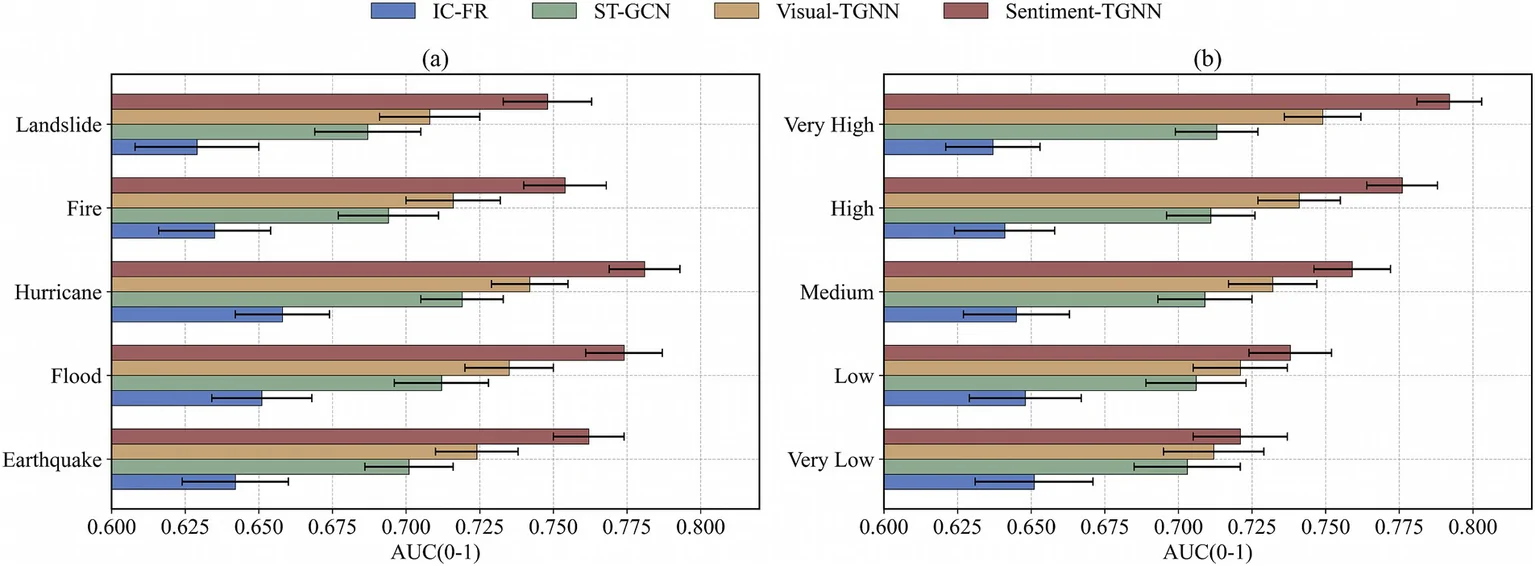
Propagation-scale AUC under disaster-type and visual-affect-intensity conditions. **(a)** Disaster categories; **(b)** visual-affect-intensity intervals. Error bars summarize repeated-run variability.

The controlled comparison evaluates how performance changes as structural dynamics, generic visual semantics, and affect-specific information are introduced. Table 5 summarizes the architectural differences and category-wise AUC ranges; Figure 5 provides the disaster-type and affect-intensity breakdown. All four systems use the same reconstructed samples and evaluation protocol.

**Table 5:** Controlled comparison of propagation-scale models under the common protocol.

Method	Graph dynamics	Generic visual input	Affect-specific input	Category-wise AUC range	Interpretive role
IC-FR	No learned dynamics	No	No	0.629–0.658	Classical cascade-feature baseline
ST-GCN	Discrete spatiotemporal graph	No	No	0.687–0.719	Tests structural dynamics
Visual-TGNN	Time-aware graph	Yes	No	0.708–0.742	Tests generic image semantics
Sentiment-TGNN	Time-aware graph	Yes	Yes	0.748–0.781	Proposed affect-aware system

A consistent performance gradient is observed. IC-FR yields AUC values of 0.629–0.658, ST-GCN improves the range to 0.687–0.719, and Visual-TGNN reaches 0.708–0.742. Sentiment-TGNN achieves 0.748–0.781 across disaster types; notably, its lowest category value exceeds the highest value of the generic Visual-TGNN range. Across affect-intensity intervals, its AUC increases from 0.721 to 0.792 while the reported standard deviation decreases from 0.016 to 0.011. The same-protocol comparison therefore supports the inference that affect-specific representation adds predictive information beyond generic visual semantics and graph structure. It does not show that affect causes wider diffusion.

### 5.2 Propagation-speed regression and error analysis

**Figure 6:**
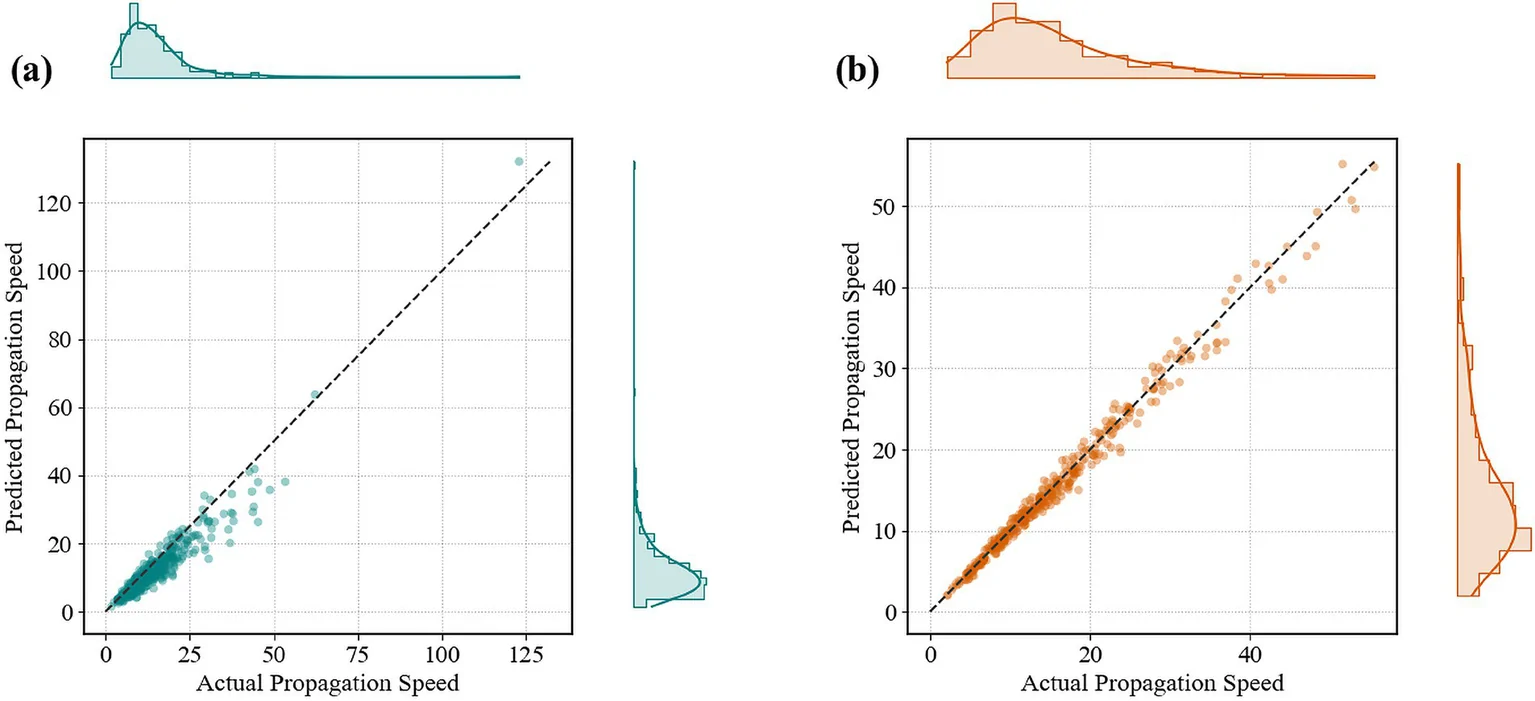
Predicted versus observed propagation speed and associated marginal distributions: **(a)** low visual-affect intensity; **(b)** high visual-affect intensity. Histograms above the *x*-axis and density curves to the right of the *y*-axis summarize the observed and predicted speed distributions, respectively.

Propagation-speed regression was examined separately for low- and high-affect-intensity samples. Figure 6 plots predicted against observed speed. Agreement with the diagonal indicates accurate estimation, while the marginal distributions reveal whether residuals are concentrated or heavy-tailed.

The low-intensity group exhibits heteroscedasticity: errors increase at higher propagation speeds and several high-speed cascades are underpredicted. This failure pattern indicates that visually ordinary images can still spread rapidly because of factors not represented by affect alone, including user influence, event salience, platform amplification, or missing parent–child links. The high-intensity group is more tightly aligned with the diagonal, suggesting that affect cues are more informative when visually salient content and rapid diffusion co-occur.

Table 6 quantifies the two conditional groups using RMSE, MAE, *R*^2^, and the prespecified comparison *p*-value. These are within-model conditional results rather than a claim that increasing affect intensity would causally accelerate diffusion.

**Table 6:** Propagation-speed regression by visual-affect-intensity group.

Sample group	RMSE (h)	MAE (h)	R-squared	*p*-value vs. low
Low visual-affect intensity	0.118	0.089	0.652	—
High visual-affect intensity	0.064	0.045	0.887	< 0.001

RMSE decreases from 0.118 to 0.064 h and MAE from 0.089 to 0.045 h in the high-intensity group, while *R*^²^ increases from 0.652 to 0.887. The reported *p*-value was < 0.001 under the prespecified group comparison. The defensible interpretation is that affective visual information improves conditional predictability in this subset; unmeasured network and event factors remain plausible explanations for the group difference.

### 5.3 Robustness across propagation stages, spatial scales, and affect intensities

**Figure 7:**
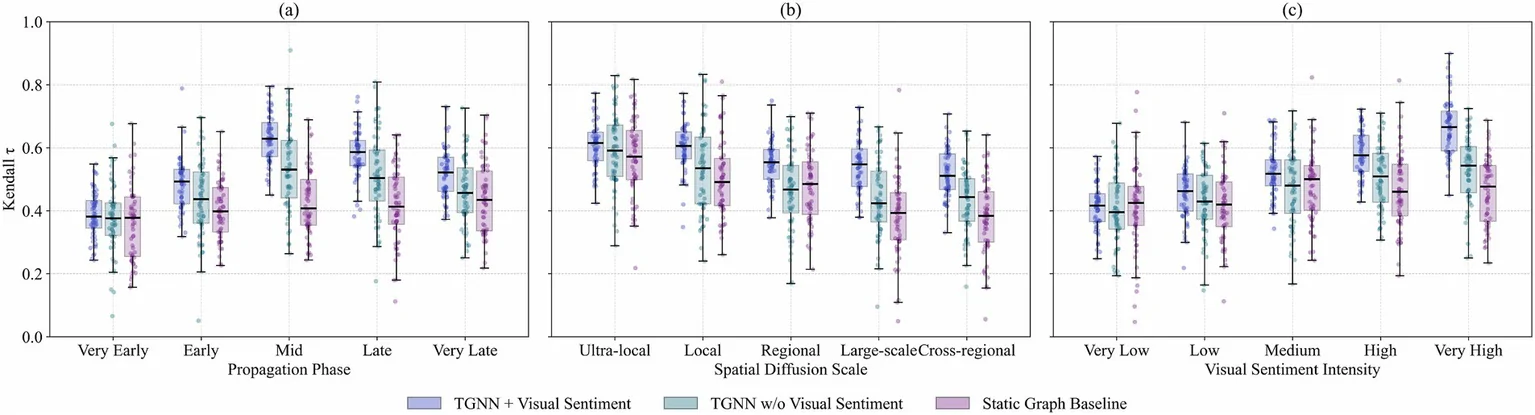
Spatial-ranking consistency across propagation stages, spatial diffusion scales, and visual-affect intensity levels: **(a)** propagation stage; **(b)** spatial diffusion scale; **(c)** visual-affect intensity.

Robustness was assessed by stratifying spatial-ranking performance across propagation stage, diffusion radius, and visual-affect intensity. Three systems were compared: the full affect-aware TGNN, the same TGNN without affect, and a static graph baseline. Figure 7 shows the resulting Kendall-tau distributions rather than only their means.

At the middle propagation stage, the full model reaches a median Kendall tau of 0.63, 0.10 above the model without affect, while the static baseline remains near 0.41 with wider dispersion. Performance declines as geographic radius expands, but under cross-regional diffusion the full model retains a median of 0.51 compared with 0.44 without affect and 0.38 for the static graph. Under very high affect intensity, the median reaches 0.67 and the variance is approximately 35% lower than in the very low condition. These patterns show that the gain is not confined to one propagation stage or one local scale.

Robustness within the reconstructed CrisisMMD protocol should not be equated with external generalizability. The cross-regional decline identifies sparse geolocation and uncertain long-distance links as important remaining error sources, and independent-event validation is still required.

### 5.4 Temporal interpretability of affect contributions

**Figure 8:**
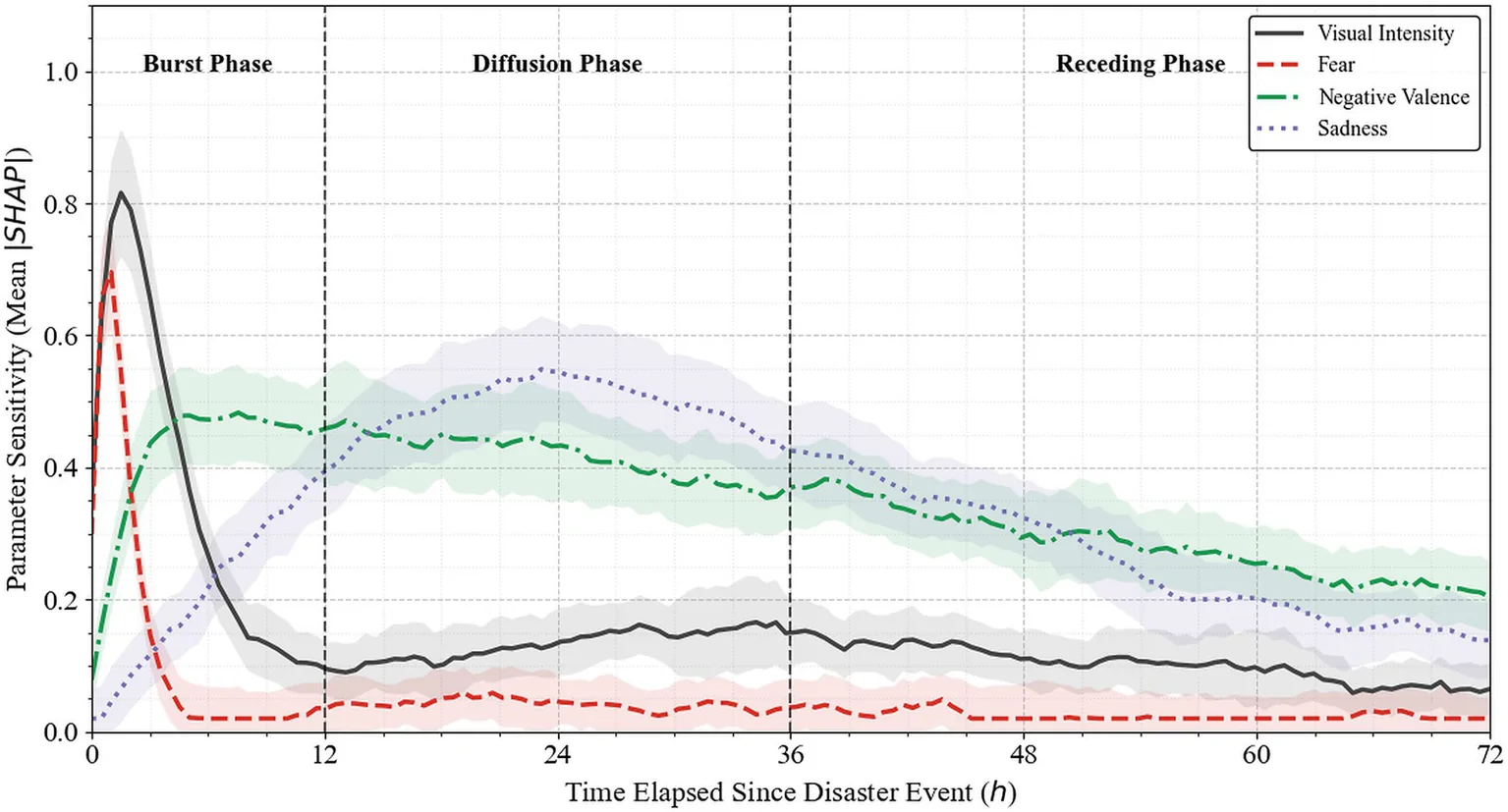
Temporal evolution of model sensitivity to visual-affect dimensions across the Burst, Diffusion, and Receding phases of disaster-image diffusion.

SHAP values were used to summarize the model sensitivity associated with visual intensity, fear, negative valence, and sadness over the 72-h observation window. The window was partitioned into burst, diffusion, and receding phases, and mean absolute contributions were aggregated with uncertainty bands. Figure 8 shows the temporal profiles.

Visual intensity and fear contribute most strongly during the outbreak phase, consistent with the model relying on highly salient early imagery. Sadness rises later during the diffusion phase, while negative valence remains comparatively stable. All affect dimensions decline during the decay phase as recency and attention diminish. The temporal sequence provides a mechanism-level explanation for the learned predictions and is consistent with the multi-stage role of affect in the graph.

SHAP values describe model sensitivity under the fitted feature distribution; they do not establish causal effects, and correlated affect dimensions can share attribution. The interpretation is therefore triangulated with the controlled baseline and ablation results rather than treated as independent proof.

### 5.5 Ablation study and statistical reliability

The ablation study removes one information source or structural constraint at a time while preserving the remaining model, data split, and training procedure. Table 7 reports the mean and standard deviation over five random seeds for all three tasks.

**Table 7:** Ablation results for visual affect and spatiotemporal constraints (mean ± standard deviation over five seeds).

Model configuration	AUC (higher)	RMSE, h (lower)	Kendall tau (higher)
Full model (affect + space–time + TGNN)	0.842 ± 0.011	0.091 ± 0.007	0.730 ± 0.018
Without visual affect	0.801 ± 0.014	0.104 ± 0.009	0.692 ± 0.021
Without temporal modeling	0.815 ± 0.013	0.137 ± 0.012	0.701 ± 0.019
Without spatial constraint	0.823 ± 0.012	0.098 ± 0.008	0.641 ± 0.026
Without space–time constraints	0.772 ± 0.016	0.162 ± 0.015	0.587 ± 0.031
Visual affect + static graph	0.789 ± 0.015	0.149 ± 0.013	0.603 ± 0.028

Each ablation removes the named component while leaving all other components unchanged. Higher AUC and Kendall tau indicate better performance; lower RMSE indicates better performance.

The full model achieves 0.842 AUC, 0.091 h RMSE, and 0.730 Kendall tau. Removing visual affect lowers AUC by 0.041, directly supporting its contribution to scale discrimination. Removing temporal modeling raises RMSE from 0.091 to 0.137 h; equivalently, the full recurrence reduces RMSE by 33.6% relative to that ablation. Removing the spatial constraint lowers Kendall tau by 0.089, confirming that geography is the principal contributor to spatial ordering. Removing both space–time constraints produces the largest overall degradation. The effect magnitudes are larger than the corresponding seed-to-seed standard deviations for the task-aligned comparisons, although no unreported *p*-value is inferred from these summaries.

### 5.6 Failure modes, computational implications, and deployment boundaries

The results identify specific failure modes rather than a uniformly solved problem. Low-affect, high-speed cascades remain difficult; cross-regional ranking declines; and incomplete parent–child links introduce topological uncertainty. These patterns imply that user influence, event salience, platform recommendation, and more complete location evidence should be incorporated before operational deployment.

The framework is an upstream affective-perception and situation-assessment layer. In a human-robot disaster workflow, predicted scale and spatial order could help an operator decide which reports deserve inspection or where additional sensing should be allocated. The model does not replace onboard sensing, localization, path planning, control, human supervision, or established emergency-management procedures.

Computational analysis indicates that sparse windowed updates are feasible, but empirical latency, energy use, device memory, and interface performance were not measured. Likewise, the reconstructed single-dataset evaluation does not demonstrate causal behavior, cross-platform generalization, or closed-loop robot benefit. These boundaries define the current evidence level and the next validation steps (Table 8).

**Table 8:** Observed failure patterns, consequences, and targeted mitigation.

Observed pattern	Likely source	Consequence	Targeted mitigation or validation
Low-affect, high-speed outliers	User influence, event salience, platform amplification	Underprediction of abrupt cascades	Add user/context features and external event signals
Cross-regional performance decline	Sparse geolocation and uncertain long-range links	Lower spatial-ranking consistency	Use denser location evidence and mobility-aware constraints
Missing parent–child relations	Incomplete platform cascade records	Topology and mechanism uncertainty	Validate on platform-native cascade logs
Single reconstructed dataset	Event and platform domain shift	Limited external generalization	Test independent disasters and platforms
No physical robot integration	Upstream analytics only	No evidence of closed-loop benefit	Simulation, operator-in-the-loop, and field experiments

### 5.7 Summary of key findings and significance

First, affect-specific representation adds information beyond generic image semantics: Sentiment-TGNN reaches 0.748–0.781 across disaster categories compared with 0.708–0.742 for Visual-TGNN, and removing affect lowers full-model AUC by 0.041. Second, temporal recurrence is the main contributor to speed estimation, as its removal increases RMSE to 0.137 h. Third, spatial constraints are essential for geographic ordering, as their removal lowers Kendall tau from 0.730 to 0.641.

The defensible inference is therefore specific: visual affect is a complementary predictive signal when integrated with time, geography, and graph structure. It is neither a stand-alone predictor nor evidence that emotional content causally drives sharing. The strongest practical significance is the conversion of an otherwise unstructured image stream into bounded, interpretable context variables that could be presented to a human-robot decision-support interface.

The main innovation is the linked evidence chain from affect representation to graph integration, controlled comparison, module-level ablation, temporal interpretation, and explicit scope limitation. External-event validation, platform-native cascades, empirical efficiency measurements, and closed-loop embodied evaluation remain necessary before deployment.

## 6 Conclusion

This study developed a visual-affect-guided spatiotemporal graph framework for modeling disaster-image diffusion as an upstream decision-support signal. Continuous affect embeddings were inserted into a conservatively reconstructed dynamic graph, updated with a time-aware recurrence, and mapped to propagation-scale, speed, and spatial-ranking outputs. On 4,450 reconstructed CrisisMMD samples, the full model achieved an AUC of 0.842 ± 0.011, an RMSE of 0.091 ± 0.007 h, and a Kendall tau of 0.730 ± 0.018. Controlled baselines and ablations showed that affect-specific representation, temporal recurrence, and spatial constraints contributed primarily to scale discrimination, speed estimation, and spatial ordering, respectively.

The findings support visual affect as an interpretable predictive feature within a spatiotemporal diffusion model. They do not establish causal influence, external cross-platform generalization, real-time device performance, or closed-loop robot benefit. Future work should validate the model on platform-native cascades and independent events, add user and context features for low-affect high-speed outliers, measure device-level efficiency, and integrate the resulting context variables with operator-in-the-loop simulation and physical human-robot experiments.

## Data Availability

The original contributions presented in the study are included in the article/supplementary material, further inquiries can be directed to the corresponding author. The source CrisisMMD dataset is available under the conditions established by its original providers. The reconstructed metadata structure, preprocessing description, and implementation code supporting this study can be made available by the corresponding author on reasonable request, subject to the source-data terms of use.
